# Performance Overview of the Latest Video Coding Proposals: HEVC, JEM and VVC

**DOI:** 10.3390/jimaging7020039

**Published:** 2021-02-22

**Authors:** Miguel O. Martínez-Rach, Héctor Migallón, Otoniel López-Granado, Vicente Galiano, Manuel P. Malumbres

**Affiliations:** Computer Engineering Department, Miguel Hernández University, 03202 Elche, Spain; hmigallon@umh.es (H.M.); otoniel@umh.es (O.L.-G.); vgaliano@umh.es (V.G.); mels@umh.es (M.P.M.)

**Keywords:** HEVC, JEM, VVC, video coding standards, performance

## Abstract

The audiovisual entertainment industry has entered a race to find the video encoder offering the best Rate/Distortion (R/D) performance for high-quality high-definition video content. The challenge consists in providing a moderate to low computational/hardware complexity encoder able to run Ultra High-Definition (UHD) video formats of different flavours (360°, AR/VR, etc.) with state-of-the-art R/D performance results. It is necessary to evaluate not only R/D performance, a highly important feature, but also the complexity of future video encoders. New coding tools offering a small increase in R/D performance at the cost of greater complexity are being advanced with caution. We performed a detailed analysis of two evolutions of High Efficiency Video Coding (HEVC) video standards, Joint Exploration Model (JEM) and Versatile Video Coding (VVC), in terms of both R/D performance and complexity. The results show how VVC, which represents the new direction of future standards, has, for the time being, sacrificed R/D performance in order to significantly reduce overall coding/decoding complexity.

## 1. Introduction

The importance of developing high-performance video codecs for the audiovisual entertainment industry is widely recognized. Rising consumption of more immersive video content with higher resolutions, from video games to video streaming delivery services, is pushing both industry and academy towards seeking new video codecs with the best possible coding performance. However, the varied and not-always-compatible facets of coding performance must be taken into account, such as higher video resolutions, higher frame rates, real-time response for 360${}^{\circ }$ video, and AR/VR immersive platforms. The High-Efficiency Video Coding (HEVC) standard [1] was initially intended to be the successor of AVC/H.264 [2]. However, it did not penetrate the industry as successfully (mainly due to licensing costs), and other alternatives promising better performance or royalty-free usage emerged [3,4]. A set of new video coding technologies is thus being proposed by the Joint Video Exploration Team (JVET), a joint ISO/IEC MPEG and ITU-VCEG initiative created to explore tools that offer video coding capabilities beyond HEVC.

The JVET team started its exploration process by implementing new coding enhancements in a software package known as the Joint Exploration Test Model (JEM) [5,6]. Its main purpose was to investigate the benefits of adding coding tools to the video coding layer. It is worth noting that JEM’s main purpose was not to establish a new standard but to identify modifications beyond HEVC that would be worthy of interest in terms of compression performance. The main goal was to achieve bit rate savings of 25–30% compared to HEVC [7]. Experimental results using the All Intra (AI) configuration [8] showed that the new model (JEM 3.0) achieved an 18% reduction in bit rate, although at the expense of a major increase in computational complexity (60x) with respect to HEVC. On the other hand, by applying a Random Access (RA) configuration, JEM obtained an average bit rate reduction of 26% with a computational complexity increment of 11x.

JEM’s increase in computational complexity with respect to HEVC was so huge that a complexity-reduction strategy had to be undertaken to compete with other emerging coding proposals. The JVET team thus decided to change the exploration process to the new Versatile Video Coding (VVC) [9,10] standard project. The main objective of VVC is to significantly improve compression performance compared to the existing HEVC, supporting the deployment of higher-quality video services and emerging applications such as 360${}^{\circ }$ omnidirectional immersive multimedia and high-dynamic-range (HDR) video.

Following JVET’s exploration to find a successor to HEVC, we need to build a deeper understanding of the key factors involved in this evolution: the Rate/Distortion (R/D) performance of new coding tools and the increase in coding complexity. Therefore, a detailed evaluation of HEVC, JEM, and VVC proposals was performed in the present study to analyze the results of this evolution.

To begin, in Section 2, we conduct a comparative analysis of the new JEM and VVC coding approaches using the HEVC as a reference. In Section 3, we present a set of experimental tests that were performed, with a detailed analysis of JEM and VVC improvements to R/D performance compared to the HEVC coding standard. The impact of new coding tools on coding complexity is also described. Conclusions are drawn in Section 4.

## 2. Overview and Comparison of Video Coding Techniques

As the JEM codec is based on the HEVC reference software (called HEVC Test Model (HM)) and the VVC standard is based on JEM, the overall architecture of the three evaluated codecs is quite similar to that of the HEVC HM codec. The three codecs thus share the hybrid video codec design. The coding stages, however, were modified in each encoder; they included modification or removal of techniques in order to improve the previous standard [9,11,12]. For example, the three codecs use closed-loop prediction with motion compensation from previously decoded reference frames or intra prediction from previously decoded areas of the current frame, but the picture partitioning schema vary for each encoder. Furthermore, the VVC standard is currently in the stage of evaluation of proposals, that is, in the “CfP results” stage, implying that the final architecture has not been definitely defined, and therefore some of the following VVC descriptions are based on currently accepted proposals [9,13]. The VVC encoder seeks a trade-off between computational complexity and R/D performance, and therefore many of the techniques included in JEM have been optimized to reduce complexity. Some have even been fully removed, specifically: mode dependent transform (DST-VII), mode dependent scanning, strong intra smoothing, hiding of sign data in transform coding, unnecessary high-level syntax (e.g., VPS), tiles and wavefronts, and finally, quantization weighting. The most relevant techniques used by the three under evaluation will be described below. They are evaluated mainly focusing in the trade-off between computational complexity and the R/D performance. Detailed information about the encoders can be found in [10,12,14] for HEVC, JEM and VVC, respectively.

### 2.1. Picture Partitioning

Picture partitioning is the way in which encoders divide each video sequence frame into a set of non-overlapping blocks. In HEVC, this partitioning is based on a quad tree structure called Coding Tree Units (CTUs) [1]. A CTU can be further partitioned into Coding Units (CUs), Prediction Units (PUs), and Transform Units (TUs). PUs store the prediction information in the form of Motion Vectors (MVs), and PU sizes range from 64×64 to 8×8 using either symmetrical or asymmetrical partitions. HEVC uses eight possible partitions for each CU size: 2Nx2N, 2NxN, Nx2N, NxN, 2NxnU, 2NxnD, nLx2N and nRx2N.

**Figure 1:**
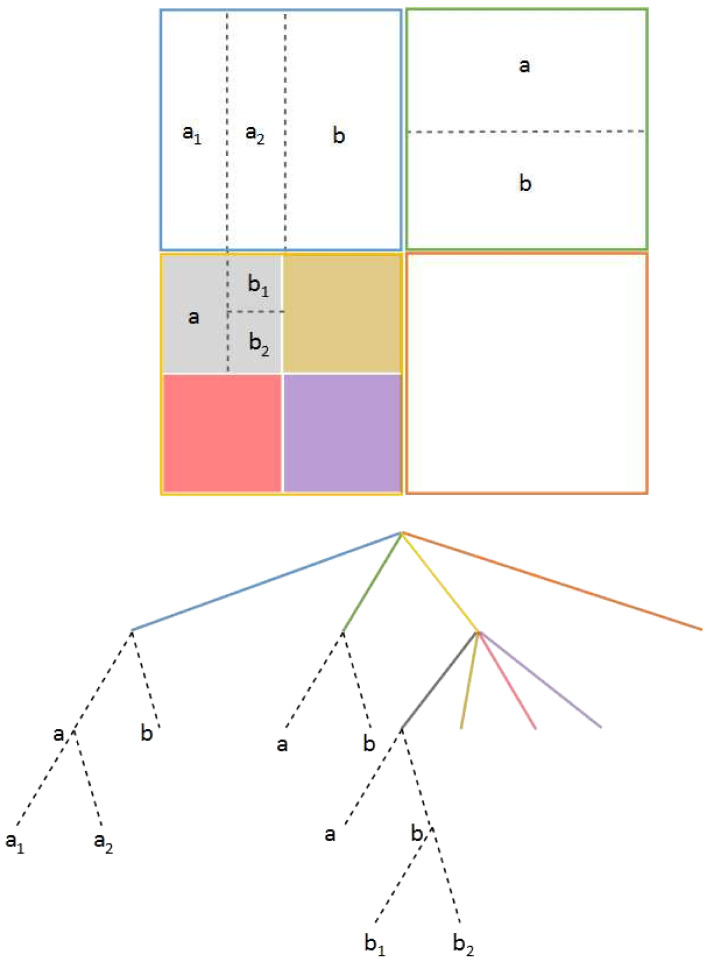
JEM and VVC QTBT Partition schema.

The picture partitioning schema is modified in JEM in order to simplify the prediction and transform stages; it should not be partitioned further, since the main partitioning schema encompasses the desired sizes for prediction and transform. The highest level is also called a CTU, as in HEVC, but the main change is that block splitting below the CTU level is performed first using a quad tree as in HEVC, and for each branch, a binary partition is made at a desired level to obtain the leaves. This partition method is called Quad Tree plus Binary Tree (QTBT). This partitioning schema offers a better match with the local characteristics of each video sequence frame so the organization in CUs, PUs, and TUs is no longer needed [15]. The leaves are considered as CUs and can have either square or rectangular shapes. The CTU can reach up to 256×256 pixels and only the first partition should be set into four square blocks. For lower partitions, the quad tree or binary tree can be used in this order. Figure 1 shows an example of a CTU partition and its quad tree plus binary tree graphical representation, where the quad tree reaches two levels (continuous colored lines), after which the binary tree starts (dotted lines labeled as a and b).

**Figure 2:**
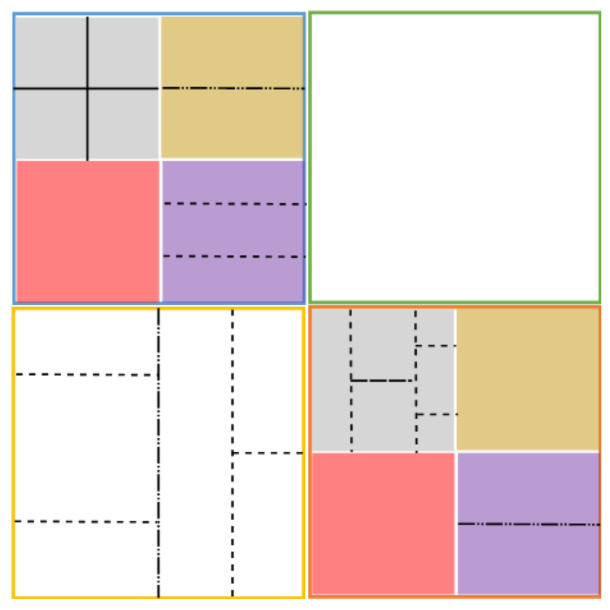
Example of QT + MTT partition for VVC.

The same QTBT partitioning schema is also used in VVC, but some of the proposed partitioning schemes are also of interest. For example, nested recursive Multi-Type Tree (MTT) partitioning is proposed: after an original quad-tree partition, a ternary or binary split can be chosen alternatively at any desired level. This new partition schema is called Quad-Tree plus Multi-Type Tree (QT + MTT) block partitioning. In Figure 2, we can see how some nodes have a ternary partition first and then a binary partition, or vice versa. The maximum CTU size is fixed at 128 × 128 pixels with variable sizes for the resulting CUs. As in the JEM encoder, these CUs are not partitioned further for transform or prediction unless the CU is too large for the maximum transform size (64×64). This means that in most cases, the CU, PU, and TU have the same size. Based on the Benchmark Set Results [16], rate savings of up to 12% on average are obtained only when using the QT-MTT instead of the QTBT, with significantly reduced encoding time. Several interesting proposals can also be found to use asymmetric rectangular binary modes and even diagonal (wedge-shaped) binary split modes.

### 2.2. Spatial Prediction

In the intra prediction stage, the JEM and VVC encoders increase the number of directional intra-modes to capture the finer edge direction presented in natural videos. The 33 directional intra-modes of the HEVC are thus increased to 65 while the planar and DC modes remain equal. All directional modes are also applied to chroma intra-prediction. To adapt to the greater number of directional intra-modes, the intra-coding method uses the six Most Probable Modes (MPMs) in JEM, while only three MPMs with additional processing and a pruning process that removes duplicated modes to be included in the MPM list are used in VVC.

Furthermore, several new coding proposals are included in both JEM and VVC with respect to HEVC to improve the intra prediction stage. Some of these proposals are improved in VVC with respect to JEM but rely on the same concepts. For example, for entropy coding of the 64 non-MPM modes, a six-bit Fixed Length Code (FLC) is used in JEM and VVC. The interpolation filter is increased from a three-tap filter (used in HEVC) to a four-tap filter. A new Cross-Component Linear Model (CCLM) prediction is also included to reduce cross-component redundancy in chroma samples. The prediction is based on the reconstructed luma samples of the same CU by using a proposed linear model. A Position Dependent Prediction Combination (PDPC) method is included. It uses unfiltered and filtered boundary reference samples, which are applied depending on the prediction mode and block size. PDPC tries to adapt to the different smoothing needed for pixels close to and far from the block borders and statistical variability when increasing the size of blocks. VVC also adaptively replaces several conventional angular intra prediction modes with wide-angle intra prediction modes for non-square blocks where the replacement depends on the blocks’ aspect ratio.

### 2.3. Temporal Prediction

In H.265/HEVC, one PU is always associated with only one set of motion information (motion vectors and reference indices). When facing inter-prediction with the new QTBT partition schema in JEM, each CU will have a maximum of one set of motion information. Two sub-CU-level motion-vector-prediction methods are included, however, that split a large CU into sub-CUs with related motion information. With the Alternative Temporal Motion Vector Prediction (ATMVP) method, each CU is split into four square sub-CUs for which motion information is obtained. In the Spatial-Temporal Motion Vector Prediction (STMVP) method, motion vectors of the sub-CUs are derived recursively by using the temporal motion vector predictor and a neighbouring spatial motion vector. In JEM, accuracy increases to 1/16 of a pixel for the internal motion vector storage and the Merge candidate, whereas one-quarter of a pixel is used for motion estimation as in HEVC. The highest level of motion vector accuracy is used in motion compensation inter-prediction for the CU coded with Skip/Merge mode.

In HEVC, only a translation motion model is applied for Motion Compensation Prediction (MCP), while in the real world, there are many kinds of motions, for example, zoom in/out, rotation, perspective motions, and other irregular motions. In order to improve motion compensation, JEM and VVC include an advanced MCP mode that uses affine transformation. The affine-transform-based motion model was adopted to improve MCP for more complicated motions such as rotation and zoom. Affine-motion estimation for the encoder uses an iterative method based on optical flow and is quite different from conventional motion estimation for translational motion models. The model builds an affine motion field composed of sub-CUs’ motion vectors, obtained by using the affine transform for the centre pixel of each sub-CU block with a precision of one-sixteenth of a pixel. The smallest CU partition is 4×4, so an 8×8 CU should be used to apply the affine model. Some proposals increase this precision up to 1/64 pixel for VVC.

Furthermore, to reduce the blocking artifacts produced by motion compensation, JEM (also inherited in VVC) uses Overlapped Block Motion Compensation (OBMC), which performs a weighted average of overlapped block segments during motion prediction. OBMC can be switched on and off using syntax at the CU level. Both encoders also include Local Illumination Compensation (LIC), which is adaptively switched on and off for each inter-mode coded CU in order to compensate local luminance variations between current and reference blocks in the motion compensation process. It is based on a linear model for luminance changes that obtains its parameters from current CU luminance values and referenced CU samples.

### 2.4. Transform Coding

For transform coding, the HEVC uses Discrete Cosine Transform (DCT-II) for block sizes over 4×4 pixels and the Discrete Sine Transform (DST-VII) for 4×4 block sizes. JEM includes a new Adaptive Multiple Transform (AMT) that uses different DCT and DST families from those used in HEVC. The specific DCT finally used for each block, whose size is below or equal to 64, is signalled by a CU-level flag. Different transforms can be applied to the rows and columns in a block. In intra mode, different sets of transforms are applied depending on the selected intra prediction mode, whereas for inter prediction, the same transforms (both vertical and horizontal) are always applied. AMT complexity is relatively high on the encoder side, since different transform candidates need to be evaluated. Several optimization methods are included in JEM to lighten this complexity.

JEM and VVC also include an intra Mode-Dependent Non-Separable Secondary Transform (MDNSST), which is defined and applied only to the low-frequency coefficients between the core transform and quantization at the encoder and between dequantization and the core inverse transform at the decoder. The idea behind the MDNSST is to improve intra prediction performance with transforms adapted to each angular prediction mode. Furthermore, JEM includes a Signal Dependent Transform (SDT) intended to enhance coding performance, taking advantage of the fact that there are many similar patches within a frame and across frames. Furthermore, such correlations are exploited by the Karhunen-Loève Transform (KLT) up to block sizes of 16.

VVC increases the TU size up to 64, which is essential for higher video resolution, for example, 1080p and 4K sequences. However, for large transform blocks (64×64), high-frequency coefficients are zeroed out so only low frequencies are retained. For example, in an M × N block, if M or N is 64, only the first 32 coefficients (left and top, respectively) are retained.

### 2.5. Loop Filter

JEM includes two new filters in addition to the deblocking filter and the sample adaptive offset present in the HEVC encoder, which remain the same but with slight configuration modifications when the Adaptive Loop Filter (ALF) is enabled. These new filters consist in the ALF with block-based filter adaptation and a Bilateral Filter (BF). The filtering process in the JEM first applies the deblocking filter followed by the Sample Adaptive Offset (SAO) and finally the ALF. Intra prediction is performed after the bilateral filtering, and the rest of the filters are applied after intra prediction. The BF is a non-linear, edge-reserving, noise-reducing smoothing filter applied by replacing the intensity of all pixels with a weighted average of intensity values from nearby pixels; it has been designed using a lookup table to minimize the number of calculations [17].

The ALF in JEM software is designed to support up to 25 filter coefficient sets that are decided after gradient calculation, that is, according to the direction and activity of local textures. A filter is selected for each 2×2 block among the 25 available filters. This aims to reduce visible artefacts such as ringing and blurring by reducing the mean absolute error between the original and the reconstructed images. In VVC, the ALF is improved with some new variants: 4×4 classification-based blocks (gradient strength and orientation) are used for luma, while the filter sizes are 7×7 for luma and 5×5 for chroma filters. A signaling flag is also included in the CTU.

### 2.6. Entropy Coding

Three improvements to the Context-based Adaptive Binary Arithmetic Coding (CABAC), the arithmetic encoder used in HEVC, are included in JEM. The first improvement is a modified model to set the context for the transform coefficients. To select the context, a transform block is split in three areas where coefficients in each area are processed in different scan passes as explained in [18]. The final selection of the context, among those assigned to each area, is determined for each coefficient depending on the values of previously scanned neighbouring coefficients. The second improvement is a multi-hypothesis probability estimation, which uses two probability estimates associated with each context model updated independently, based on the probabilities obtained before and after decoding each specific bin. The final probability used in the interval subdivision of the arithmetic encoder is the average of these two estimations. Finally, the third improvement relies on the models’ adaptive initialization, where instead of using fixed tables for context model initialization as in HEVC, initial probability states for inter-coded slices can be initialized by inheriting the statistics from previously coded pictures.

## 3. Comparative Analysis between HEVC, JEM and VVC

In this section, we present a comparative analysis of R/D (following guidelines stated in documents [19,20]) and encoding time overhead between HEVC, JEM, and VVC encoding standards using the AI, Low Delay (LD), Low Delay P (LDP), and RA coding modes. Under the AI coding mode, each frame in the sequence is coded as an independent (I) frame, so no temporal prediction is used, i.e., no frame use information from other frames. When LD and LDP coding modes are used, only the first frame is encoded as an I frame, and all subsequent frames are split into multiple image groups (Group Of Pictures, GOP), coded as B (LD coding mode) or P (LDP coding mode) frames, in both modes information from other frames are used, but a P frame has only one reference list of frames while a B frame has two reference lists. Under RA coding mode the frames are also divided into GOPs, but an I-frame is inserted for an integer number of GOPs and the coding order of the frames differs from the playing order, coding order preserved in the rest of coding modes.

The platform was an HP Proliant SL390 G7 of which only one of the Intel Xeon X5660 processors was used and the compiler was GCC v.4.8.5 [21]. Thirty-three video sequences with different resolutions were used in our study and are listed in Table 1. Detailed information about the test video sequences can be found, for example, in [22], and they can be downloaded from ftp://ftp.tnt.uni-hannover.de/pub/svc/testsequences (accessed on 23 March 2015). The reference software for the encoders was HM 16.3 [23] for HEVC and JEM 7.0 [12] for JEM and VTM 1.1 for VVC [9,10], using their default configurations except for the HEVC encoder, where the Main10 Profile was chosen in order to work with the same colour depth as the rest of the encoders.

The Bjontegaard-Delta rate (BD-rate) metric [24] represents the percentage bit-rate variation between two sequences encoded with different encoding proposals with the same objective quality. A negative value implies an improvement in coding efficiency, that is, a lower rate required to encode with the same quality, between one proposal and another. Table 2, Table 3, Table 4, Table 5 and Table 6 show the BD rate obtained when comparing the coding efficiencies of JEM and VVC with respect to HEVC for each of the coding modes. Each table corresponds to video sequences that share the same frame resolution.

After analyzing the results provided in Table 2, Table 3, Table 4, Table 5 and Table 6, we can observe rate savings (negative BD-rate values) for each frame resolution and that both the JEM and the VVC encoder outperform the HEVC encoder. Rate savings with respect to HEVC amount to an average of 32.81% for JEM but only 16.08%, on average, for VVC. Maximum rate savings in our tests were obtained when using the RA coding mode: up to 39.04% for JEM and 22.87% for VVC.

The results provided in Table 2, Table 3, Table 4, Table 5 and Table 6 and the average values for each frame resolution, shown in Table 7, lead us to conclude that frame resolution does not affect the results for rate savings. Therefore, the average for all sequences, regardless of their resolution, is also presented in Table 7. Regarding the coding mode, different coding modes can be observed to provide different rate savings. Performance decreased as expected in this order: RA, LDP, LD, and AI; that is, the best rate savings were obtained when using RA and lower rate savings were obtained when using the AI coding mode. These results were also obtained independently for the frame resolution.

As shown, JEM provided better performance than VVC in all cases. The average values in Table 7 (for all images) allow us to obtain the relative performances of JEM and VVC shown in Table 8, where the third column represents the number of times that JEM improves VVC in terms of R/D performance (BD-Rate). As mentioned earlier, JEM outperformed VVC in terms of rate savings in all encoding modes, but not to the same extent for each one. As shown in Table 8, JEM is on average almost four times better than VVC in AI coding mode, while it is only two times better in RA coding mode. These results should be compared with those obtained for the computational time needed to process the sequences in each mode.

Table 9 shows as the computational time, in seconds, for one video sequence per resolution. As can be seen, the computational cost increase of both JEM and VVC with respect to HEVC is really significant. Table 10, Table 11, Table 12, Table 13, Table 14, show the computational time increase, expressed as a percentage, with respect to HEVC for each Quantization Parameter (QP) value and coding mode. As expected, less computational time is required in all coding modes as the QP parameter increases. The increase in computational time depends on the scene content and not on the scene resolution.

The JEM encoder requires considerably more time to encode in any coding mode, but this increase is extremely high in the AI coding mode. For some sequences in our test, up to 6,419% more time is required than with HEVC. In the LP, LDP, and RA modes, the increase was also very high. These results show that all the techniques included in JEM to provide better R/D results actually bring about much more computational complexity.

In the VVC encoder, some of these techniques were removed from the reference software as a trade-off between computational complexity and R/D performance, and many others were improved to reduce the time overhead. This can be seen in Table 10, Table 11, Table 12, Table 13, Table 14 when comparing the results for the JEM and VVC columns. In all cases, the time overhead of VVC with respect to HEVC is lower than that of JEM. As the negative values show for many sequences, VVC needs even less time to encode than the HEVC, especially in the case of higher QP values. This reduction achieved by VVC reaches up to 76% compared to HEVC when using the LD coding mode for the SlideEditing (1280 × 720) sequence for a QP value of 37.

Regarding the time results obtained in the LP, LDP, and RA coding modes, we analysed which mode had statistically less time overhead with respect to HEVC. We could thus compare the time overheads of LD, LDP, and RA by conducting Friedman’s rank test [25], making it possible to determine which coding mode leads to statistically less computing overhead. The test’s output includes the *p*-value, a scalar value in the [0…1] range, which, when below 0.05, indicates that the results are statistically relevant, and the $\xi^{2}$ value, which expresses the variance of the mean ranks. Friedman’s rank test was applied to data in the columns LD, LDP, and RA for VVC in Table 10, Table 11 and Table 12, obtaining a mean rank of 1.18 for LD, 2.13 for LDP, and 2.69 for RA, with a p-value of $5.17\times 10^{-34}$ and $\xi^{2}$ = 135.29. The AI mode undoubtedly introduces the highest computational overhead, note that considering the rest of the modes (LD, LDP and RA) and as the results were statistically significant, it can be concluded that the LD coding mode introduces, statistically, less overhead for VVC when using the default software configuration, while RA generates the highest overhead for VVC.

**Figure 3:**
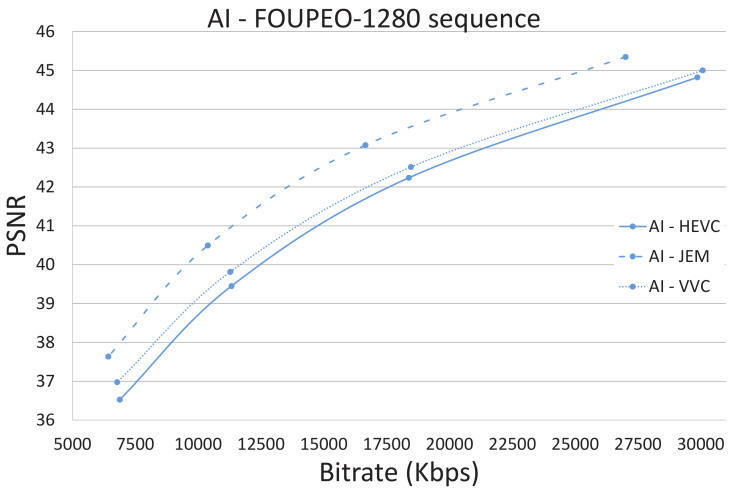
All intra: HEVC, JEM and VVC comparison.

**Figure 4:**
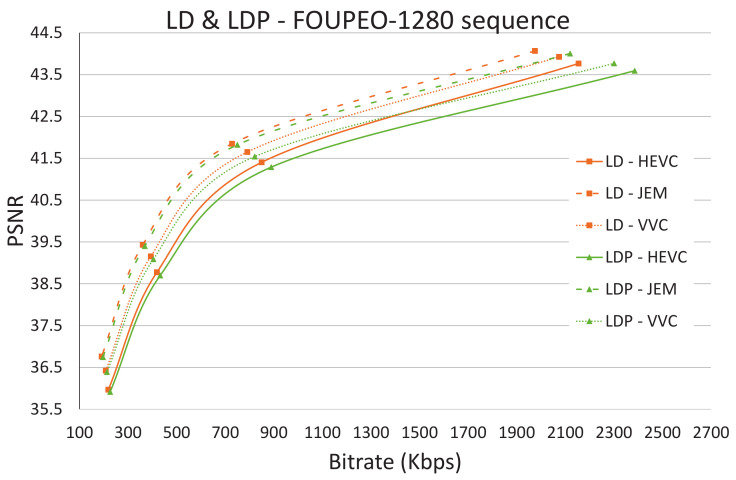
Low Delay B and Low Delay P: HEVC, JEM and VVC comparison.

**Figure 5:**
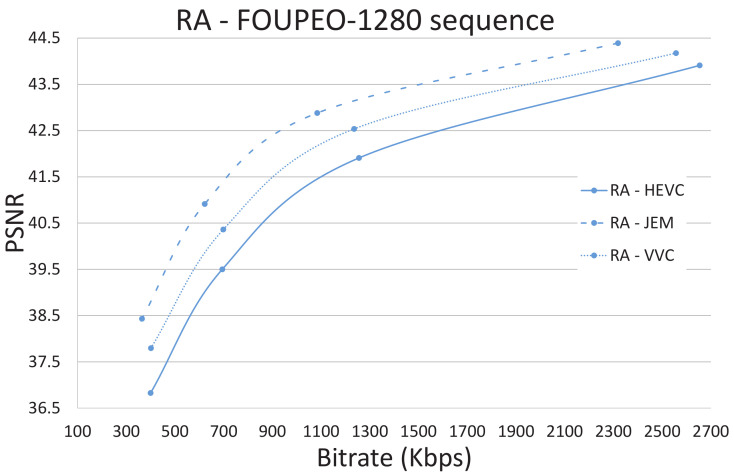
Random access: HEVC, JEM and VVC comparison.

Figure 3, Figure 4 and Figure 5 show the R/D performance obtained using the three encoders HEVC, JEM, and VVC for the FourPeople 1280 × 720 sequence. Figure 3 shows the results for the AI coding mode, Figure 4 shows those for the LD and LDP coding modes, and Figure 5 shows those for the RA coding mode. The figures illustrate how the JEM encoder clearly outperforms HEVC and VVC in terms of R/D, as revealed in Table 2, Table 3, Table 4, Table 5 and Table 6 above; that is, the R/D curve for JEM is clearly better than the two other curves for all the coding modes and sequences. However, this improvement comes at the expense of a much greater amount of computational time. In the same way, VVC also outperforms HEVC in terms of R/D in all scenarios and even, as observable in Table 10, Table 11 and Table 12, in terms of computational time for many sequences.

For example, in the case of the FourPeople 1280 × 720 sequence (see Figure 5 and Table 13), if we focus on the LD mode and on the lowest QP value (highest rate), VVC needs 15% less computational time than HEVC, although it obtains a lower rate and better Peak Signal-to-Noise Ratio (PSNR). JEM obtains a better R/D curve with these settings but at the cost of a 238% increase in computational time compared to HEVC.

## 4. Conclusions

In this paper, we summarized the evolution of the JVET exploration process to propose a new video coding standard that significantly improves the performance of HEVC. We took into account, however, further design factors such as coding complexity. We performed an exhaustive experimental study to analyze the behavior of JEM and VVC video coding projects in terms of coding performance and complexity.

The results showed that VVC achieves a better trade-off between R/D performance and computational effort, and as shown for many sequences, takes even less coding time than HEVC when using the LD, LDP, and RA coding modes.

Nevertheless, in the AI coding mode, the increase in complexity was still too high in the case of VVC and overwhelming in the case of JEM. VVC needs to improve its coding tools to achieve a better trade-off between coding performance and complexity in the AI mode. The standard is currently not closed and some proposals may come forward in this direction. Efforts should be made to define coding tools that are effective in terms of performance while offering a low-complexity design or at least a straightforward parallelization process.

Given the rise in video resolutions and low-latency video (VR/AR, 360${}^{\circ }$, etc.) demands, future coding standards should be cleverly designed to broadly support different application requirements and to better use available hardware resources.

The experimental study presented made it possible to discern which techniques to improve coding standards can be definitively applied, with the improvement of R/D not the only factor to be taken into account. In addition, the increase in bandwidth of current networks is not sufficient for the increases in bit rates due to the increase in video resolutions, quality, and different flavours (360${}^{\circ }$, AR/VR, etc.).

## Figures and Tables

**Table 1:** Sequences and its related information grouped by resolution.

Resolution	Sequence	Frame Rate	Num Frames	Time (s)
416 × 240	BasketballPass	50	500	10
	BlowingBubbles	50	500	10
	BQSquare	60	600	10
	Flowervase 416 × 240	30	300	10
	Keiba	30	300	10
	Mobisode2	30	300	10
	RaceHorses	30	300	10
832 × 480	BasketballDrill	50	500	10
	BasketballDrillText	50	500	10
	BQMall	60	600	10
	Flowervase	30	300	10
	Keiba	30	300	10
	Mobisode2	30	300	10
	PartyScene	50	500	10
	RaceHorses	30	300	10
1280 × 720	Johnny	60	600	10
	KristenAndSara	60	600	10
	FourPeople	60	600	10
	SlideEditing	30	300	10
	SlideShow	20	500	25
	Vidyo1	60	600	10
	Vidyo3	60	600	10
	Vidyo4	60	600	10
1920 × 1080	BasketballDrive	50	500	10
	BQTerrace	60	600	10
	Cactus	50	500	10
	Kimono1	24	240	10
	ParkScene	24	240	10
	Tennis	24	240	10
2560 × 1600	NebutaFestival	60	300	5
	PeopleOnStreet	30	150	5
	SteamLocomotiveTrain	60	300	5
	Traffic	30	150	5

**Table 2:** 416 × 240: BD-rate between JEM and VVC with respect to HEVC.

Sequence	AI		LD		LDP		RA	
416 × 240	JEM	VVC	JEM	VVC	JEM	VVC	JEM	VVC
BasketballPass	−17.92	−5.77	−22.42	−10.60	−23.87	−10.57	−28.74	−12.47
BlowingBubbles	−14.46	−1.93	−21.34	−7.17	−22.98	−32.09	−30.18	−14.19
BQSquare	−12.71	−1.13	−31.18	−4.82	−34.64	−5.33	−36.17	−13.53
Flowervase	−14.22	−3.65	−31.91	−7.40	−32.09	−7.95	−34.73	−16.90
Keiba	−15.80	−3.66	−20.03	−10.84	−22.88	−11.61	−25.24	−15.02
Mobisode2	−19.51	−10.43	−32.61	−15.79	−34.38	−16.22	−28.76	−17.74
RaceHorses	−16.97	−2.86	−20.56	−8.38	−21.66	−8.68	−26.69	−11.14

**Table 3:** 832 × 480: BD-rate between JEM and VVC with respect to HEVC.

Sequence	AI		LD		LDP		RA	
832 × 480	JEM	VVC	JEM	VVC	JEM	VVC	JEM	VVC
BasketballDrill	−30.77	−6.33	−28.55	−12.04	−30.14	−12.32	−37.35	−17.68
BasketballDrillText	−29.88	−7.29	−29.29	−13.84	−31.96	−13.71	−37.38	−19.11
BQMall	−19.55	−5.54	−23.73	−11.52	−26.91	−12.09	−32.93	−15.51
Flowervase	−16.05	−4.20	−30.04	−11.59	−31.93	−11.99	−37.55	−18.65
Keiba	−19.13	−6.82	−23.62	−14.06	−26.32	−15.19	−31.29	−21.33
Mobisode2	−24.76	−11.55	−39.53	−20.84	−41.52	−21.62	−37.46	−22.27
PartyScene	−14.82	−2.32	−22.89	−7.98	−25.31	−7.85	−32.27	−15.01
RaceHorses	−15.66	−2.78	−19.47	−7.52	−22.07	−7.91	−25.93	−10.65

**Table 4:** 1280 × 720: BD-rate between JEM and VVC with respect to HEVC.

Sequence	AI		LD		LDP		RA	
1280 × 720	JEM	VVC	JEM	VVC	JEM	VVC	JEM	VVC
Johnny	−22.76	−7.27	−30.79	−14.44	−36.50	−16.29	−37.62	−18.77
KristenAndSara	−22.71	−4.83	−30.62	−14.69	−33.68	−16.46	−36.73	−17.35
FourPeople	−22.39	−5.82	−26.13	−13.91	−29.11	−15.01	−36.25	−17.96
SlideEditing	−15.24	−4.63	−18.87	−9.26	−18.69	−8.67	−17.34	−7.82
SlideShow	−21.67	−5.39	−31.98	−13.92	−32.69	−13.62	−33.92	−17.81
Vidyo1	−22.57	−6.79	−28.19	−13.27	−31.46	−14.85	−37.36	−18.47
Vidyo3	−21.00	−6.83	−31.99	−14.73	−38.78	−16.17	−39.04	−19.67
Vidyo4	−20.26	−6.10	−27.57	−14.28	−31.24	−15.49	−35.85	−19.25

**Table 5:** 1920 × 1080: BD-rate between JEM and VVC with respect to HEVC.

Sequence	AI		LD		LDP		RA	
1920 × 1080	JEM	VVC	JEM	VVC	JEM	VVC	JEM	VVC
BasketballDrill	−21.93	−7.89	−27.75	−14.80	−32.31	−15.87	−35.17	−16.39
BQTerrace	−16.90	−2.64	−23.18	−8.29	−34.41	−9.04	−31.25	−12.09
Cactus	−19.09	−4.46	−28.79	−11.24	−32.33	−12.38	−37.03	−14.04
Kimono1	−17.91	−3.83	−18.72	−8.76	−23.50	−10.79	−27.06	−12.07
PartyScene	−16.94	−1.49	−16.47	−8.07	−18.86	−8.88	−29.21	−14.84
Tennis	−22.93	−9.60	−30.72	−20.58	−33.53	−20.54	−34.12	−22.87

**Table 6:** 2560 × 1600: BD-rate between JEM and VVC with respect to HEVC.

Sequence	AI		LD		LDP		RA	
2560 × 1600	JEM	VVC	JEM	VVC	JEM	VVC	JEM	VVC
PeopleOnStreet	−22.68	−4.07	−25.54	−10.65	−27.95	−11.38	−33.13	−12.99
SteamLocomotiveTrain	−17.76	−2.23	−27.15	−12.10	−38.48	−13.39	−31.82	−13.61
Traffic	−21.28	−4.49	−23.51	−11.73	−27.20	−12.77	−34.42	−17.39

**Table 7:** Average BD-rate for each sequence resolution and overall average for all sequences.

	AI		LD		LDP		RA	
	JEM	VVC	JEM	VVC	JEM	VVC	JEM	VVC
416 × 240	−15.94	−4.21	−25.72	−9.29	−27.50	−13.21	−30.07	−14.43
832 × 480	−21.33	−5.85	−27.14	−12.42	−29.52	−12.83	−34.02	−17.53
1280 × 720	−21.07	−5.96	−28.27	−13.56	−31.52	−14.57	−34.26	−17.14
1920 × 1080	−19.29	−4.99	−24.27	−11.96	−29.16	−12.92	−32.31	−15.39
2560 × 1600	−20.57	−3.60	−25.40	−11.49	−31.21	−12.51	−33.12	−14.67
Average	−19.63	−5.15	−26.41	−11.85	−29.67	−13.33	−32.81	−16.08

**Table 8:** Delta BD-rate between JEM and VVC.

Delta BD-Rate	JEM	VVC	JEM vs. VVC
All Intra (AI)	−19.63	−5.15	3.81
Low Delay (LD)	−26.41	−11.85	**2.23**
Low Delay P (LDP)	−29.67	−13.33	**2.23**
Random Access (RA)	−32.81	−16.08	2.04

**Table 9:** Computational times in seconds for one sequence per resolution.

	QP	AI	LD	LDP	RA
		BasketballPass 416 × 240			
HEVC	22	527	2431	1939	1812
	27	465	2140	1644	1552
	32	411	1882	1395	1336
	37	361	1683	1214	1190
JEM	22	29,964	26,371	15,545	22,718
	27	23,080	21,440	12,433	17,970
	32	17,009	18,519	10,306	14,748
	37	11,683	15,527	8436	11,822
VVC	22	5409	4849	3773	4582
	27	5073	3661	2868	3530
	32	4386	2828	2185	2766
	37	3732	2084	1624	2024
		BasketballDrill 832 × 480			
HEVC	22	2210	9078	7144	6584
	27	1857	7761	5800	5559
	32	1609	6696	4836	4808
	37	1425	5949	4192	4368
JEM	22	109,421	79,465	47,261	68,793
	27	83,040	71,445	40,846	57,227
	32	56,868	60,640	33,929	46,495
	37	36,767	50,840	27,232	37,275
VVC	22	23,876	18,084	14,207	16,599
	27	20,794	13,668	10,676	12,479
	32	17,686	9962	7721	9292
	37	13,963	7025	5488	6840
		Johnny 1280 × 720			
HEVC	22	4538	15,403	10,554	10,827
	27	4040	13,554	8829	9720
	32	3753	12,892	8288	9373
	37	3529	12,450	7997	9188
JEM	22	151,216	61,812	36,623	52,826
	27	102,630	38,261	22,208	34,482
	32	72,343	29,035	17,399	28,172
	37	49,344	24,498	14,680	24,919
VVC	22	34,762	15,348	12,203	10,222
	27	29,338	7474	5640	5513
	32	26,339	4907	3653	3990
	37	21,873	3452	2535	3175
		BasketballDrive 1920 × 1080			
HEVC	22	10,244	48,610	38,564	34,528
	27	8181	39,663	29,779	28,113
	32	7337	34,796	25,286	24,968
	37	6751	31,661	22,291	22,909
JEM	22	567,635	512,247	322,412	414,611
	27	322,769	353,861	212,822	269,029
	32	193,253	277,098	158,824	208,452
	37	123,278	229,743	127,396	168,444
VVC	22	103,497	102,281	79,889	101,284
	27	85,268	66,788	52,304	66,966
	32	70,865	47,079	37,134	50,806
	37	57,536	35,171	27,800	37,843
		PeopleOnStreet 2560 × 1600			
HEVC	22	6130	31,619	24,847	23,262
	27	5315	26,697	20,157	19,558
	32	4851	23,746	17,341	17,036
	37	4371	21,715	15,518	15,406
JEM	22	345,329	238,260	164,760	221,201
	27	262,107	167,359	109,198	173,224
	32	180,976	155,175	96,291	143,041
	37	125,464	135,855	82,466	122,144
VVC	22	61,212	66,931	55,174	68,658
	27	56,757	45,810	37,368	53,447
	32	50,428	40,452	31,730	44,645
	37	42,662	33,252	24,351	36,243

**Table 10:** Resolution 2560 × 1600: Computational time increase compared to HEVC for each QP and coding mode.

Sequence		AI		LD		LDP		RA	
2560 × 1600	QP	JEM	VVC	JEM	VVC	JEM	VVC	JEM	VVC
PeopleOnStreet	22	5533%	899%	654%	112%	563%	122%	851%	195%
	27	4831%	968%	527%	72%	442%	85%	786%	173%
	32	3630%	939%	553%	70%	455%	83%	740%	162%
	37	2770%	876%	526%	53%	431%	57%	693%	135%
SteamLocomotive Train	22	3638%	700%	1046%	136%	853%	139%	1140%	225%
	27	2441%	636%	711%	47%	554%	67%	755%	110%
	32	1743%	569%	528%	−1%	412%	17%	559%	53%
	37	1252%	486%	401%	−31%	312%	−15%	434%	12%
Traffic	22	5310%	950%	434%	57%	317%	53%	561%	67%
	27	4430%	942%	341%	16%	279%	17%	469%	25%
	32	3454%	920%	290%	−10%	236%	0%	387%	−2%
	37	2641%	892%	213%	−36%	174%	−29%	299%	−25%

**Table 11:** Resolution 416 × 240: Computational time increase compared to HEVC for each QP and coding mode.

Sequence		AI		LD		LDP		RA	
416 × 240	QP	JEM	VVC	JEM	VVC	JEM	VVC	JEM	VVC
BasketballPass	22	5581%	926%	985%	99%	702%	95%	1154%	153%
	27	4863%	991%	902%	71%	656%	74%	1058%	127%
	32	4043%	968%	884%	50%	639%	57%	1004%	107%
	37	3137%	934%	823%	24%	595%	34%	893%	70%
BlowingBubbles	22	6419%	913%	782%	102%	529%	99%	898%	103%
	27	6163%	935%	691%	60%	490%	62%	843%	72%
	32	5490%	986%	638%	29%	465%	37%	788%	45%
	37	4710%	1048%	553%	−6%	401%	3%	671%	9%
BQSquare	22	6219%	874%	516%	92%	354%	76%	637%	75%
	27	5566%	900%	410%	38%	297%	28%	527%	19%
	32	4965%	926%	303%	−6%	246%	−2%	442%	−12%
	37	4323%	928%	245%	−36%	200%	−31%	346%	−36%
Flowervase	22	4354%	935%	597%	41%	376%	43%	642%	13%
	27	3571%	880%	475%	−6%	317%	−4%	505%	−19%
	32	2986%	853%	377%	−29%	265%	−24%	429%	−35%
	37	2512%	830%	317%	−49%	227%	−44%	392%	−48%
Keiba	22	4956%	843%	914%	75%	671%	74%	1,076%	123%
	27	4430%	855%	837%	49%	621%	55%	998%	98%
	32	3548%	861%	776%	28%	571%	34%	941%	74%
	37	2679%	821%	703%	6%	530%	16%	809%	42%
Mobisode2	22	3026%	883%	633%	63%	454%	74%	694%	83%
	27	2143%	756%	556%	27%	382%	41%	569%	39%
	32	1601%	709%	476%	1%	333%	12%	501%	8%
	37	1217%	613%	403%	−24%	302%	−12%	446%	−14%
RaceHorses	22	6141%	912%	1078%	121%	748%	111%	1180%	168%
	27	5357%	960%	958%	86%	680%	88%	1078%	143%
	32	4838%	1058%	925%	62%	643%	65%	1062%	122%
	37	3790%	1047%	890%	38%	634%	47%	980%	87%

**Table 12:** Resolution 832 × 480: Computational time increase compared to HEVC for each QP and coding mode.

Sequence		AI		LD		LDP		RA	
832 × 480	QP	JEM	VVC	JEM	VVC	JEM	VVC	JEM	VVC
BasketballDrill	22	4852%	981%	775%	99%	562%	99%	945%	152%
	27	4372%	1020%	821%	76%	604%	84%	930%	124%
	32	3435%	999%	806%	49%	602%	60%	867%	93%
	37	2480%	880%	755%	18%	550%	31%	753%	57%
BasketballDrillText	22	4867%	958%	780%	93%	563%	96%	937%	146%
	27	4529%	1020%	828%	74%	614%	79%	938%	121%
	32	3719%	994%	827%	50%	602%	56%	874%	92%
	37	2906%	910%	738%	19%	542%	32%	773%	59%
BQMall	22	5443%	947%	763%	67%	531%	64%	883%	99%
	27	4715%	965%	723%	38%	503%	39%	795%	69%
	32	3999%	986%	654%	13%	459%	19%	709%	43%
	37	3058%	947%	604%	−8%	423%	−1%	620%	17%
Flowervase	22	4033%	895%	660%	49%	434%	49%	777%	53%
	27	3241%	834%	570%	6%	381%	11%	620%	12%
	32	2573%	767%	508%	−15%	348%	−14%	530%	−13%
	37	1961%	679%	384%	−41%	262%	−36%	412%	−37%
Keiba	22	5023%	827%	976%	79%	739%	80%	1148%	145%
	27	4080%	806%	875%	51%	669%	59%	1012%	110%
	32	3097%	792%	782%	28%	598%	36%	881%	81%
	37	2183%	736%	695%	7%	516%	14%	772%	53%
Mobisode2	22	2617%	778%	627%	65%	450%	75%	673%	93%
	27	1762%	668%	503%	24%	361%	38%	509%	44%
	32	1174%	540%	432%	−2%	301%	11%	421%	10%
	37	806%	426%	358%	−24%	256%	−12%	358%	−15%
PartyScene	22	6165%	873%	704%	99%	506%	95%	802%	116%
	27	5883%	949%	625%	60%	465%	61%	767%	87%
	32	5361%	1011%	588%	35%	455%	45%	727%	62%
	37	4580%	1060%	538%	6%	413%	18%	628%	28%
RaceHorses	22	5784%	883%	1075%	131%	776%	121%	1197%	200%
	27	5251%	948%	918%	87%	655%	84%	1096%	165%
	32	4374%	984%	940%	68%	680%	74%	1064%	143%
	37	3199%	926%	810%	32%	590%	42%	969%	105%

**Table 13:** Resolution 1280 × 720: Computational time increase compared to HEVC for each QP and coding mode.

Sequence		AI		LD		LDP		RA	
1280 × 720	QP	JEM	VVC	JEM	VVC	JEM	VVC	JEM	VVC
Johnny	22	3232%	666%	301%	0%	247%	16%	388%	−6%
	27	2440%	626%	182%	−45%	152%	−36%	255%	−43%
	32	1827%	602%	125%	−62%	110%	−56%	201%	−57%
	37	1298%	520%	97%	−72%	84%	−68%	171%	−65%
KristenAndSara	22	3642%	759%	410%	19%	325%	28%	479%	25%
	27	2864%	715%	293%	−22%	232%	−14%	337%	−15%
	32	2163%	667%	227%	−44%	182%	−37%	271%	−36%
	37	1583%	591%	174%	−60%	138%	−54%	221%	−50%
FourPeople	22	4305%	892%	339%	23%	273%	33%	454%	29%
	27	3536%	855%	238%	−15%	199%	−5%	333%	−6%
	32	2891%	812%	188%	−36%	156%	−29%	274%	−25%
	37	2226%	750%	157%	−50%	131%	−44%	227%	−40%
SlideEditing	22	4248%	705%	129%	−69%	110%	−62%	271%	−46%
	27	4020%	697%	120%	−72%	105%	−66%	254%	−52%
	32	3746%	737%	123%	−74%	99%	−70%	238%	−56%
	37	3388%	707%	120%	−76	95%	−72%	226%	−59%
SlideShow	22	2346%	506%	370%	−19%	313%	−8%	499%	16%
	27	1943%	459%	347%	−27%	292%	−16%	459%	2%
	32	1654%	413%	332%	−36%	276%	−26%	425%	−9%
	37	1362%	351%	308%	−43%	251%	−34%	395%	−19%
Vidyo1	22	4135%	930%	321%	18%	254%	26%	437%	20%
	27	3158%	919%	247%	−16%	200%	−8%	319%	−15%
	32	2284%	826%	202%	−40%	156%	−33%	257%	−34%
	37	1710%	703%	152%	−54%	127%	−48%	214%	−48%
Vidyo3	22	3506%	838%	402%	23%	319%	35%	496%	30%
	27	2770%	805%	283%	−23%	226%	−11%	353%	−15%
	32	2135%	728%	224%	−45%	172%	−39%	273%	−37%
	37	1569%	622%	180%	−59%	136%	−53%	224%	−50%
Vidyo4	22	4034%	889%	480%	22%	369%	32%	552%	31%
	27	3096%	847%	339%	−23%	262%	−14%	394%	−12%
	32	2272%	787%	268%	−45%	208%	−39%	314%	−32%
	37	1635%	683%	212%	−60%	161%	−54%	255%	−48%

**Table 14:** Resolution 1920 × 1080: Computational time increase compared to HEVC for each QP and coding mode.

Sequence		AI		LD		LDP		RA	
1920 × 1080	QP	JEM	VVC	JEM	VVC	JEM	VVC	JEM	VVC
BasketballDrill	22	5441%	910%	954%	110%	736%	107%	1101%	193%
	27	3845%	942%	792%	68%	615%	76%	857%	138%
	32	2534%	866%	696%	35%	528%	47%	735%	103%
	37	1726%	752%	626%	11%	472%	25%	635%	65%
BQTerrace	22	5510%	716%	695%	97%	574%	100%	801%	105%
	27	4704%	816%	409%	11%	316%	22%	556%	19%
	32	3608%	806%	309%	−29%	244%	−18%	388%	−25%
	37	2610%	763%	212%	−55%	163%	−48%	280%	−48%
Cactus	22	5914%	880%	872%	97%	593%	97%	891%	133%
	27	4468%	874%	640%	55%	485%	57%	710%	94%
	32	3369%	870%	612%	25%	460%	37%	613%	67%
	37	2382%	792%	501%	3%	372%	16%	514%	36%
Kimono1	22	4199%	733%	720%	95%	583%	99%	843%	156%
	27	3055%	710%	595%	53%	460%	63%	703%	112%
	32	2294%	702%	574%	22%	419%	33%	588%	77%
	37	1590%	656%	500%	2%	361%	7%	477%	37%
PartyScene	22	5836%	862%	500%	67%	378%	64%	661%	89%
	27	4915%	891%	430%	29%	336%	34%	566%	49%
	32	3782%	874%	412%	2%	321%	9%	483%	20%
	37	2684%	810%	321%	−25%	251%	−15%	381%	−7%
Tennis	22	3596%	825%	1025%	118%	805%	119%	1150%	215%
	27	2508%	780%	871%	77%	673%	88%	958%	164%
	32	1665%	667%	729%	43%	553%	56%	874%	130%
	37	1166%	568%	711%	23%	539%	36%	778%	90%

## Data Availability

Contact with corresponding author.
